# Research on Gas Pipeline Leakage Prediction Model Based on Physics-Aware GL-TransLSTM

**DOI:** 10.3390/biomimetics10110743

**Published:** 2025-11-05

**Authors:** Chunjiang Wu, Haoyu Lu, Dianming Liu, Chen Wang, Jianhong Gan, Zhibin Li

**Affiliations:** 1 School of Software Engineering, Chengdu University of Information Technology, Chengdu 610225, China; 2 Sichuan Province Engineering Technology Research Center of Support Software of Informatization Application, Chengdu 610225, China; 3 School of Automation Engineering, University of Electronic Science and Technology of China, Chengdu 611731, China; 4 School of Information and Software Engineering, University of Electronic Science and Technology of China, Chengdu 610054, China

**Keywords:** natural gas pipeline leakage, deep learning, GL-TransLSTM, physics-informed gated attention, adaptive sliding window, time series forecasting

## Abstract

Natural gas pipeline leak monitoring suffers from severe environmental noise, non-stationary signals, and complex multi-source variable couplings, limiting prediction accuracy and robustness. Inspired by biological perceptual systems, particularly their multimodal integration and dynamic attention allocation, we propose GL-TransLSTM, a biomimetic hybrid deep learning model. It synergistically combines Transformer’s global self-attention (emulating selective focus) and LSTM’s gated memory (mimicking neural temporal retention). The architecture incorporates a multimodal fusion pipeline; raw sensor data are first decomposed via CEEMDAN to extract multi-scale features, then processed by an enhanced LSTM-Transformer backbone. A novel physics-informed gated attention mechanism embeds gas diffusion dynamics into attention weights, while an adaptive sliding window adjusts temporal granularity. This study makes evaluations on an industrial dataset with methane concentration, temperature, and pressure, GL-TransLSTM achieves 99.93% accuracy, 99.86% recall, and 99.89% F1-score, thereby significantly outperforming conventional LSTM and Transformer-LSTM baselines. Experimental results demonstrate that the proposed biomimetic framework substantially enhances modeling capacity and generalization for non-stationary signals in noisy and complex industrial environments through multi-scale fusion, physics-guided learning, and bio-inspired architectural synergy.

## 1. Introduction

In modern industrial systems, pipeline networks are vital for energy distribution, especially in oil, gas, petrochemical, and nuclear sectors, where safety is critical [1,2,3,4,5,6,7,8,9]. Leaks can cause severe environmental damage, public safety hazards, and cascading impacts: (1) massive energy waste, (2) explosion risks, and (3) major economic losses [10,11,12,13]. In aging infrastructure, leakage exceeds 40% of total water transport in some regions [14], and over 130 deaths in China in 2013 are linked to pipeline failures. High-pressure conditions (e.g., several MPa from backfill slurry) further increase risks of pipe rupture, joint failure, or equipment malfunction [15,16], thus leading to soil/road contamination, costly remediation, operational disruptions (e.g., in mining backfilling) and secondary accidents. Consequently, real-time pipeline monitoring systems are of significant engineering value, enabling early anomaly detection, prompt response, and the mitigation of safety-related and economic consequences [17].

In intelligent fault diagnosis, detection performance critically depends on the discriminative power of extracted features. Dimensionality reduction improves both computational efficiency and identification accuracy. Traditional methods such as mass/volume balance [18] and pressure point analysis (PPA) [19] lack adaptability despite their simplicity. Deep learning (DL) overcomes these limitations through powerful end-to-end feature learning [20,21,22], gaining prominence in nondestructive testing [21,23]. CNNs have been widely explored: Kang et al. [24] achieve 99.3% leak recognition using CNN-SVM; Liu’s team [25] propose a robust CNN-TL model; and Ahmad et al. [26] apply 1D-CNN for leak size estimation though ignoring flow regime variations. Specifically, CNNs bypass manual feature engineering and struggle with temporal dynamics.

LSTM with gating-based memory mechanism excels in modeling time series data, which has improved leak detection performance [27]. Li et al. [28] obtain over 90% accuracy in buried pipeline monitoring. However, extensive methods prioritize spatial features and underutilize temporal evolution, limiting spatiotemporal fusion. Recent advances address this gap: the LSS model [29] integrates LSTM with statistical process analysis for bearing degradation prediction; Zuo et al. [30] develop a semi-supervised LSTM autoencoder framework to reduce label dependency; Yang and Zhao [31] employ BiLSTM to differentiate leaks from pressure disturbances with potential loss of subtle features. Ravikumar et al. [32] propose MDRL-SLSTM, which combines CNN and residual learning, and stacks LSTM layers for gearbox health monitoring. Qiao et al. [33] present a dual-input CNN-LSTM model that fuses time- and frequency-domain features, thus achieving high accuracy, robustness, and adaptability under variable loads and noise.

Although big data-driven machine learning can model complex nonlinear relationships, it remains challenged by high-dimensional data, vanishing gradients, and exploding gradients. Long Short-Term Memory (LSTM) networks, widely used for sequential and multi-source heterogeneous data, excel at capturing temporal patterns and offer robust performance [34]. The architecture inspired by biological neural systems employs gating mechanisms, which emulate selective memory and forgetting, reflecting biomimetic intelligence. Nevertheless, conventional LSTMs still suffer from limited feature extraction and attenuation of long-term dependencies in very long or highly dynamic sequences.

Inspired by multimodal information integration and attention allocation mechanisms in biological perceptual systems, deep learning models continue to emulate principles of natural intelligence. For instance, primates dynamically allocate attentional resources in complex environments, preferentially processing sensory inputs most critical for decision-making a “selective focus” strategy that underpins the global self-attention mechanism in the Transformer architecture. This biological insight enables Transformers to dispense with sequential temporal dependencies and instead leverage parallel computation to efficiently capture global interdependencies among high-dimensional features, substantially enhancing both prediction accuracy and training efficiency [35]. In contrast, Long Short-Term Memory (LSTM) networks partially mimic biological memory through gated mechanisms that regulate the retention and forgetting of temporal information, and their inherently sequential processing limits their capacity to model long-range dependencies. Critical information often attenuates or is lost during propagation through extended sequences.

Natural intelligence arises from attentional modulation in individual nervous systems and the coordinated interaction of multi-level; heterogeneous units are exemplified by swarm intelligence in bee colonies, and complex group behaviors emerge from simple, decentralized interactions. This principle has inspired hybrid modeling approaches that combine distinct architectures to leverage complementary strengths and overcome the limitations of single-model designs [36]. Accordingly, by integrating LSTM’s temporal modeling capacity with the Transformer’s biologically inspired attention mechanism, it represents a dual biomimetic strategy mimicking both temporal memory and selective perception in biological systems. This fusion embodies the core idea of biomimetic integrated intelligence, where heterogeneous components collaborate to achieve robust, adaptive, and efficient intelligent behavior.

To address technical limitations like insufficient feature extraction, information loss in standalone LSTM models, and suboptimal detection accuracy due to manual hyper-parameter selection, this study proposes a novel physics-informed GL-TransLSTM model. The primary innovations of the proposed model are threefold:

- Proposing Physics-Aware Gated Attention (PAGA), which converts physical variables into gating signals to dynamically adjust self-attention weights. This enhances sensitivity to weak leakage signals and incorporates causal reasoning by focusing on key physical events.
- Designing Physics-Guided Adaptive Sliding Window (PG-ASW) with a Differentiable Window Controller (DWC) that dynamically optimizes window length, position, and feature weights based on real-time parameters. It enhances handling of non-stationary, multi-scale industrial data and aligns data with physical processes.
- Developing the GL-TransLSTM model: a hybrid architecture integrating CEEMDAN decomposition is an improved Transformer with PAGA and positional encoding. It acquires multi-scale feature extraction, long-range dependency capture, and robust prediction in high-noise industrial environments.

## 2. Related Work

### 2.1. LSTM

Long Short-Term Memory (LSTM) proposed by Hochreiter in 1997 [37] is a specialized recurrent neural network (RNN) architecture designed for processing sequential, time-dependent data. By incorporating memory units and a gating mechanism, comprising input, forget and output gates, LSTM effectively controls information flow, enabling selective retention, updating, and filtering of data across time steps. This structure mitigates gradient vanishing/exploding issues, enhancing training stability and scalability. Thanks to its ability to capture long-range dependencies, LSTM excels in time series prediction and has become a widely used and powerful model in this domain [38].

**Figure 1:**
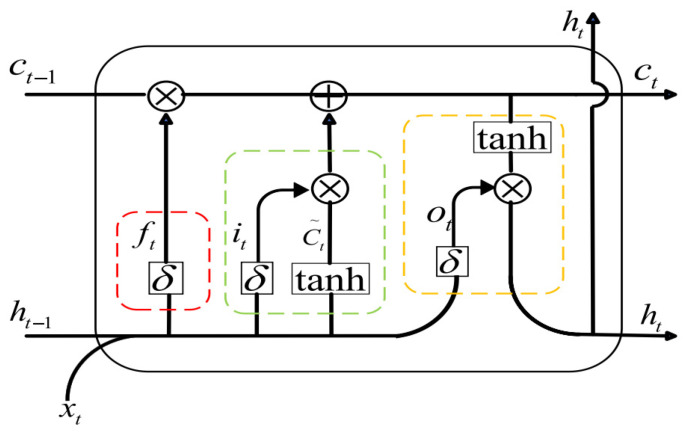
Architecture diagram of LSTM.

The LSTM network consists of multiple LSTM layers, which contain multiple LSTM units. Each unit includes an input gate, a forget gate, and an output gate, along with a candidate state computation structure, and the gates interconnected via corresponding neurons. The hidden state of a LSTM layer is the output produced by its memory units at each time step, thus combining cell state information and being modulated by the output gate. This hidden state is updated at every time step. The key point to the LSTM lies in its use of these three gates, which is used to dynamically control the memory state of the cell. Moreover, a single LSTM unit is shown in Figure 1.

The memory cell consists of a forgetting gate ($f_{t}$), an input gate ($i_{t}$), an output gate ($o_{t}$), and a cell state ($c_{t}$). At time *t*, the three gates receive the input $x_{t}$ at the current time point and the output $h_{t-1}$ at the previous time point. The function of forgetting Gate $f_{t}$ is to decide which information from the previous time point is discarded and update the retained information to the state $c_{t}$ at the current time point. To achieve this function, the forgetting gate usually uses the sigmoid activation function to limit the output result to the range of 0 to 1. This numerical range has a variable amplitude similar to the switch, which also enables the network to switch flexibly between completely discarding the stored information and retaining all the contents. $f_{t}$ is the output to measure the degree of information retention. When value of $f_{t}$ is 0, which completely discards the previous information, the value of 1 is used to completely retain the past content. The input gate ($i_{t}$) is responsible for managing the new information to be incorporated into the current unit state. The process can be divided into two steps: the first step is to use the sigmoid function to evaluate whether the update conditions are met. The second step is to generate temporary candidate memory content (${\tilde{C}}_{t}$) through the tanh function. The output gate ($o_{t}$) determines which information in the unit state is used for output. Furthermore, the definition of the three door units is given by the following formula.

(1) $$f_{t}=\sigma (W_{f}x_{t}+W_{f}h_{t-1}+b_{f}),$$

(2) $$i_{i}=\sigma (W_{i}x_{i}+W_{i}h_{t-1}+b_{i}),$$

(3) $$o_{t}=\sigma (W_{o}x_{t}+W_{o}h_{t-1}+b_{o}),$$

(4) $$\tilde{C_{t}}=\tanh(W_{a}x_{t}+W_{a}h_{t-1}+b_{a}),$$

where *W* is used to define the weight matrix in the calculation formula; b is used to define the offset vector in the calculation formula; σ is used to define the sigmoid function in the calculation formula; $x_{t}$ represents the input at the current time step; and $h_{t}$ represents the specific output at the current time step.

As far as LSTM neural network is concerned, it receives time series data as input, and it sends elements one by one and processes in sequence, in a different way from the traditional network, because once all of them are injected at one time, time correlation is often difficult to retain. When arriving at the time node *t*, the flow of information in the network unit is divided into several steps, which together constitute mechanisms such as forgetting and updating, and also adjust around the output signal.

1. In the cell state $c_{t-1}$ at time *t*−1, it is necessary to determine which content must be discarded, and the forgetting gate value calculated according to Equation (1) becomes the main reference, which is between 0 and 1. The closer the value is to 1, the more information is forgotten, and only a small part of the information is deleted.

2. For the updated cell state $c_{t}$, it is necessary to obtain the candidate value by Equation (4), and then combine the input gate value obtained by Equation (2) to evaluate the component size that can be added to $c_{t}$ [39]. When the gate value tends toward 1, the amount of information is relatively increased; otherwise only a limited part is included.

3. Combining steps 1 and 2, we obtain the neuron state $c_{t}$ at the current time point:

(5) $$c_{t}=f_{t}\cdot c_{t-1}+i_{t}\cdot {\tilde{C}}_{t},$$

(6) $$h_{t}=o_{t}\cdot \tanh(c_{t}),$$

Among them, tanh is the activation function. The values of $f_{t}$, $i_{t}$, $o_{t}$, and $c_{t}$ achieve range between 0 and 1, while $h_{t}$ obtains range between −1 and 1.

4. Under the current time node, the information to be transmitted from the LSTM unit can be calculated by Equation (3), and the value is still between 0 and 1. When the value is higher, the external available content is more sufficient; otherwise the output is more limited. Finally, the actual output value at the moment is obtained by combining Equation (6).

LSTM neural network can extract and learn useful information patterns in continuous cycle sequence processing, and output refined content. Although it generates an output signal at each processing time, the final prediction analysis can be carried out only on the results of the last time step for a specific prediction task.

### 2.2. Transformer

The Transformer is a deep learning architecture designed for processing sequential data, whose core mechanism relies on self-attention to capture long-range dependencies within the input sequence, thereby achieving superior predictive performance. A standard Transformer network typically consists of multiple identical encoder or decoder layers, with the self-attention module being the most critical component. Specifically, after the input sequence is augmented with positional encodings, it undergoes linear transformations through three learnable weight matrices, commonly denoted as $\omega^{Q}$, $\omega^{K}$, and $\omega^{V}$, to produce three distinct vector representations: ***Queries*** (*Q*), ***Keys*** (*K*), and ***Values*** (*V*). The attention scores, which reflect the pairwise relevance between sequence elements, are then computed by taking the dot product between *Q* and $K^{T}$; higher scores indicate stronger dependencies, while lower scores imply weaker associations. To mitigate the effect of large dot-product magnitudes, particularly in high-dimensional spaces, the scores are scaled by the square root of the key dimension $d_{k}$. The resulting scaled attention weights are subsequently applied to the value vectors to generate the final attention output, as formalized in Equation (7):

(7) $$A(Q,K,V)=softmax\left(\frac{QK^{T}}{\sqrt{d_{k}}}\right),$$

**Figure 2:**
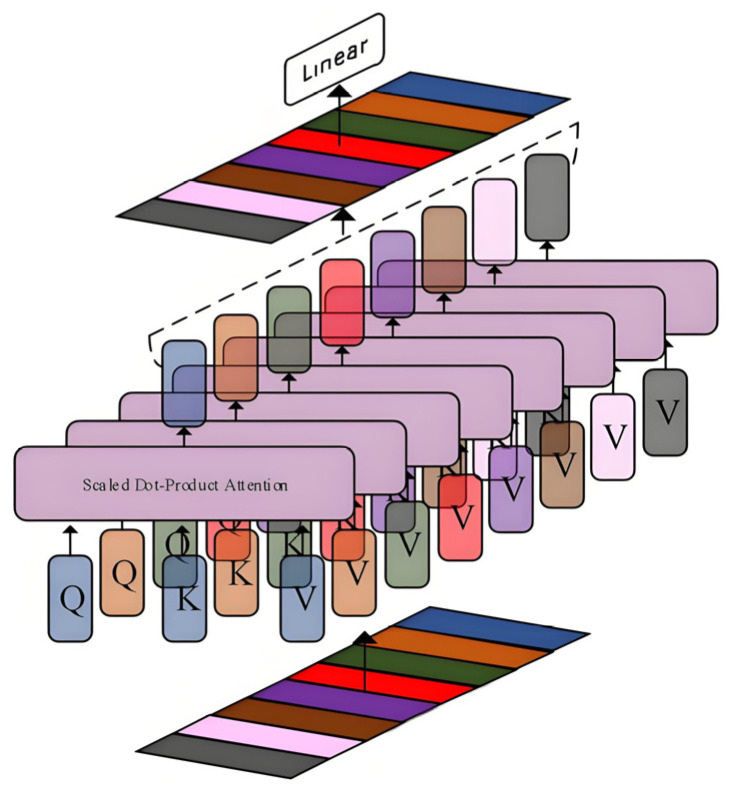
Multi-Head Attention Mechanism.

To capture features at multiple levels of abstraction, practical implementations typically employ multiple sets of distinct *Q*, *K*, and *V* matrices, each producing its own attention distribution and corresponding output representation. This design forms the Multi-Head Attention mechanism, which is illustrated in Figure 2. Specifically, *h* independent sets of *Q*, *K*, and *V* matrices are projected through separate self-attention layers, yielding *h* distinct output matrices. These outputs are then concatenated along the feature dimension and linearly transformed into a single vector whose dimensionality matches that of the original input. By performing multiple parallel attention computations with different learned projections, the multi-head mechanism enables the model to depend on diverse representation subspaces and varying levels of granularity, thereby enhancing its capacity to model complex, heterogeneous relationships within the input sequence.

### 2.3. Deep Learning for Pipeline Leakage Detection

In the field of intelligent diagnosis technology, the detection efficiency of data-driven systems essentially depends on the ability of the extracted fault features to represent the differences in various faults. By reducing the data dimension, the feature extraction process can improve the efficiency of the algorithm, which also significantly enhances the accuracy of fault identification. The traditional detection method is mainly based on the principle of mass or volume balance, and then its core can determine whether there is leakage by calculating the difference in mass or volume flow at both ends of the pipeline [18]. In contrast, the pressure point analysis (PPA) technology provides a more simplified solution, which realizes fault diagnosis by comparing the real-time monitored flow rate or pressure data with the historical statistical trajectory [19]. The advantage of PPA method is that it makes full use of the trend characteristics of historical data and realizes more informative evaluation through comparative analysis. However, the traditional diagnosis model generally has the problem of insufficient adaptability. In particular, it is worth mentioning that deep learning (DL) technology has made a breakthrough in nondestructive testing [21,23]. Among them, convolutional neural networks (CNNs) are widely concerned because of its excellent performance. Kang and other scholars [24] innovatively propose a hybrid leak detection method combining CNN and support vector machine (SVM), and the experimental results show that the recognition accuracy reached 99.3%. Professor Liu et al. [25] develop a CNN-TL pipeline leakage detection technology, which is suitable for multiple working conditions. Ahmad research group [26] use one-dimensional CNN architecture to detect the leakage size of fluid pipeline. The research shows that this method has some significant advantages in the accuracy of leakage size identification, while it could not consider the impact of different flow modes on the detection results. Although CNN technology can construct end-to-end classifiers without complex feature engineering, it still has some limitations in time series modeling. Among many variants of recurrent neural networks (RNNs), the long-term and short-term memory network (LSTM) is the most prominent because of its unique memory mechanism. The latest research data shows that the leakage detection method based on LSTM has achieved significant improvement in many indicators [27]. Li et al. [28] adopt LSTM technology to detect the leakage state of buried pipelines; then, the accuracy of the proposed method is stable above 90%. However, these methods mainly focus on the spatial distribution characteristics of data in the process of signal feature extraction, and lack of mining the dynamic evolution rules of time dimension, which reflects its limitations in multi-dimensional feature fusion. Detailed method comparison is shown in Table 1.

The LSS model [29] proposed by Professor Liu et al. creatively integrates the advantages of long-term and short-term memory (LSTM) network and process statistical analysis, which is specially used for multi-level performance degradation fault prediction of aero-engine bearings. A large number of experimental data show that the prediction accuracy of this method is significantly better than that of the traditional recurrent neural network (RNN), support vector regression, and standard LSTM model. Zuo et al. propose an innovative semi supervised leak detection framework [30], which contains two key modules—an improved LSTM automatic encoder network and a class of support vector machines—thus significantly reducing the dependence on traditional leak detection methods. Dr. Yang and Professor Zhao jointly develop a pipeline leakage detection system based on two-way long- and short-term memory network (BiLSTM) [31], which can identify leakage signals; it also effectively distinguishes typical non leakage pressure disturbances. However, it should be pointed out that these methods are still insufficient in the extraction of some specific signal features, which may lead to the loss of key information. Ravikumar research group propose a multi-scale deep residual learning fault diagnosis model (mdrl-slstm) [32], which uses stacked long-term and short-term memory networks to process sequential data in the health prediction of internal combustion engine gearbox. Firstly, the model achieves local feature extraction and dimension reduction through CNN and residual learning technology, which is verified by the gearbox vibration dataset and shows excellent diagnostic performance. Professor Qiao et al. propose a dual-input fusion model based on CNN and LSTM [33]. The developed model creatively uses time-domain and frequency-domain features to realize end-to-end fault diagnosis. The experimental results show that the model obtains high fault recognition rate, which also acquires excellent robustness and adaptability under the condition of variable load and noise interference.

## 3. GL-TransLSTM Model

In the context of industrial gas leakage monitoring, traditional Transformer models indicate strong capabilities in modeling global dependencies, the quadratic time complexity of their self-attention mechanism (O($n^{2}$) with respect to input sequence length *n*) leads to significant computational costs when processing high-frequency sensor data. Taking biogas concentration prediction as an example, the mixed release process of methane and biological gases is influenced by coupled multi-physical factors such as equipment operational status, ambient temperature and humidity, pressure gradients, wind speed, and direction. This results in complex nonlinear dynamic characteristics. When traditional Transformer models handle such data, adopting a long historical window drastically increases computational resource consumption, thus making it difficult to meet real-time requirements in industrial settings. Furthermore, transformers are prone to the “attention dilution” phenomenon when capturing short-range dynamic changes, where the model may overlook local abrupt features by focusing on global information. Early signals of gas leakage frequently manifest as local temporal patterns.

More importantly, the gas leakage process is inherently a dynamical system constrained by physical laws. For instance, according to Fick’s law of diffusion, gas concentration decays exponentially with distance; based on the ideal gas law, temperature changes directly affect gas volume and sensor readings. However, the standard Transformer model is a purely data-driven architecture and lacks the ability to explicitly encode such prior physical knowledge. Directly applying a generic Transformer to gas leakage prediction would require the model to “relearn” these fundamental laws from massive datasets, leading to inefficient training and limited generalization performance. For example, during critical physical events such as sudden temperature drops or valve openings, traditional models may fail to promptly capture the causal relationship between relevant historical segments and current leakage, thereby compromising prediction accuracy.

### 3.1. Physics-Aware Gated Attention Mechanism

To address the aforementioned challenges, this paper proposes a Physics-Aware Gated Attention (PAGA) mechanism. Its core idea is to incorporate physical variables (e.g., ambient temperature, air pressure) as gating signals to dynamically adjust the distribution of attention weights, enabling the model to emphasize the influence of critical physical events while maintaining global contextual awareness. The implementation involves three specific steps:

1. Gating Vector Generation

A small fully connected network is used to map physical variables (rate of temperature change ∆T, pressure gradient ∆P) into gating vectors $g_{t}$:

(8) $$g_{t}=\sigma (W_{g}\cdot [\Delta T_{t},\Delta P_{t}]+b_{g}),$$

where σ denotes the sigmoid function, ensuring that the gating values lie within the interval (0, 1); $W_{g}$ and $b_{g}$ represent learnable parameter matrices and bias terms, respectively. This gating vector reflects the regulatory demand of the current physical state on the attention distribution; for instance, increasing the attention weight on historical low-temperature data segments when a sudden temperature drop occurs.

2. Attention Weight Adjustment

A gating term is introduced into the standard multi-head self-attention mechanism. The modified attention weights are calculated as follows:

(9) $$a_{t,j}^{k}=softmax\left(\frac{(Q_{t}W_{k}^{Q}){\left(K_{i}W_{k}^{K}\right)}^{T}}{\sqrt{d_{k}}}\cdot g_{t}^{k}\right),$$

where *k* denotes the index of the attention head, $g_{t}^{k}$ represents the gating value of the k-th head, $Q_{t}{,K}_{i}$ corresponds to the query and key vectors, $W_{k}^{Q}{,W}_{k}^{K}$ denotes a learnable projection matrix, and $d_{k}$ indicates the dimensionality of the attention head. This design enables the model to automatically enhance attention to relevant historical segments when critical physical events occur, such as a sudden temperature drop or valve opening, thereby improving its capability to identify leakage precursors. For instance, when the valve state transitions from 0 to 1, the gating vector value increases significantly, causing the attention weights to concentrate on segments before and after the valve operation. This allows the model to clearly discern the causal relationship between “valve opening” and the subsequent concentration rise.

3. Based on Physical Priors: Attention Coupling and Regularization

To further constrain the model to learn attention distributions consistent with physical principles, a physical relevance prior matrix *C* is defined, whose elements $C_{ij}$ are computed based on the heat conduction equation and Fick’s laws of diffusion:

(10) $$C_{ij}=\exp(-\frac{\left|t_{i}-t_{j}\right|}{\tau })\cdot I(\left|\vec{r_{i}}-\vec{r_{j}}\right|<\delta ),$$

where τ denotes the diffusion time constant, reflecting the rate of gas concentration decay over time, and I is an indicator function that determines whether the distance between spatial locations ${\vec{r}}_{i}$ and ${\vec{r}}_{j}$ is below the interaction threshold δ. This matrix is embedded as prior knowledge into the gating network to guide the model in learning temporal delays and spatial diffusion characteristics. To quantify the consistency between the physical prior and the learned attention matrix *A*, an attention consistency loss is defined:

(11) $$L_{phys}=\frac{1}{T^{2}}\sum_{i,j}{\left\|A_{i,j}-C_{i,j}\right\|}^{2},$$

where *T* denotes the sequence length. This loss term is optimized jointly with the main objective during training, encouraging the model to both fit the observed data and adhere to underlying physical principles. From an information-theoretic perspective, the physical gating mechanism equips the model with the capacity for causal reasoning, enabling it to distinguish between statistical correlations and physically grounded causal relationships. For instance, the model can identify physical causal chains such as “a decrease in temperature leads to an increase in gas solubility, which in turn induces short-term fluctuations in concentration,” rather than merely interpreting temperature and concentration changes as synchronous phenomena.

The Physics-Aware Gated Attention (PAGA) mechanism significantly enhances the interpretability and robustness of gas leakage prediction models by explicitly encoding physical laws. Unlike conventional Transformer models, which typically rely on massive datasets to implicitly capture underlying physical patterns often resulting in lower training efficiency and heightened sensitivity to noise, the PAGA mechanism leverages the synergistic interaction between gating vectors and physical prior matrices. This enables the model to actively concentrate on historical segments that conform to physical causality, thereby reducing its reliance on irrelevant information. For instance, in scenarios involving valve-opening events, conventional models may produce false leakage risk assessments due to the presence of historically similar yet physically distinct operations in the training data. In contrast, PAGA utilizes the sensitivity of its gating vectors to changes in valve states, allowing it to accurately differentiate between normal operations and genuine leakage precursors. Furthermore, the incorporation of a physical relevance prior matrix provides the model with a rational initial distribution of attention weights from the early stages of training, which accelerates convergence and promotes more stable optimization.

### 3.2. Physics-Informed Adaptive Window Optimization Algorithm

In time series forecasting tasks, the sliding window technique serves as a classical data preprocessing approach to convert sequential data into a supervised learning format. However, traditional fixed-length sliding windows exhibit significant limitations when applied to non-stationary, multi-scale industrial data, such as in gas leakage prediction. Firstly, the selection of window length presents a fundamental trade-off: industrial processes (e.g., intermittent fermentation or periodic residue discharge) often exhibit both minute-level rapid fluctuations and hour-level slow variations. A fixed-length window proves inadequate in capturing such multi-scale dynamics, an overly short window fails to capture long-term dependencies (e.g., the gradual influence of temperature control strategies), while an excessively long window increases computational burden, which introduces irrelevant historical information. Secondly, conventional methods treat all features (e.g., concentration, temperature, pressure) equally, disregarding the distinct dominance of different physical variables across time scales. For instance, the rate of concentration change may be more critical during valve opening events, whereas ambient temperature becomes more influential during steady-state operation. This undifferentiated treatment leads to inefficient information utilization. Most importantly, gas leakage processes are governed by physical principles such as mass conservation and heat conduction. Window segmentation should, therefore, align with the dynamic characteristics of the physical system. Traditional methods frequently contain outliers or ignore physical consistency, resulting in erroneous predictions. Thus, there is a pressing need for an adaptive window mechanism capable of dynamically adjusting window length, position and feature combinations to achieve deep integration between data representation and underlying physical processes.

#### 3.2.1. Adaptive Window Generator

The core of the framework is a Differentiable Window Controller (DWC), which aims to generate an optimal window configuration $\left(w_{t},s_{t},f_{t}\right)$ for each prediction time step *t*, where $\left(w_{t},s_{t},f_{t}\right)$ includes the window length $w_{t}$, the start offset $s_{t}$, and a feature selection vector $f_{t}$.

The input to the DWC consist of current and recent physical states, denoted as $z_{t}=[\Delta C_{t},\Delta T_{t},{valve_{-}hange}_{t}]$, where $\Delta C_{t}=C_{t}-C_{t-1}$ represents the concentration variation, $∆T_{t}$ the temperature change, and ${valve_{-}hange}_{t}$ a binary flag indicating valve state transitions. The DWC employs a compact LSTM network to process the historical state sequence $Z_{t-k:t}$, and subsequently outputs the adaptive window parameters:

(12) $$(w_{t},s_{t},f_{t})=DWC(Z_{t-k:t};\theta_{dwc}),$$

where $\theta_{dwc}$ denotes the learnable parameters of the DWC.

To enable end-to-end training, the window parameters must be applied to the input data in a differentiable manner. This is achieved through a soft selection mechanism. For the window length $w_{t}$, a continuous weight distribution $\alpha_{t,\tau }$ is defined, where $\tau \in [t-w_{max},t]$:

(13) $$\alpha_{t,\tau }=\exp(-\frac{{\left(\tau -t+w_{t}/2\right)}^{2}}{2\sigma_{w}^{2}})$$

The mean of this Gaussian distribution is located at $t-w_{t}/2$; the standard deviation $\sigma_{w}$ controls the smoothness of the window edges. Similarly, for the feature selection vector $f_{t}\in (0,1{)}^{d}$, each element represents the retention probability of the corresponding feature. The final weighted input sequence is computed as

(14) $$\tilde{X_{t}}=\sum_{\tau =t-w_{\max}}^{t}\sum_{j=1}^{d}\alpha_{t,\tau }f_{t}\cdot x_{\tau ,j}\cdot e_{j},$$

where $e_{j}$ denotes the unit vector of the *j*-th feature,$x_{\tau ,j}$ denotes the input vector. This representation can be directly processed by standard neural networks.

#### 3.2.2. Physics-Guided Adaptive Window Constraint Mechanism

To prevent the DWC from generating window configurations that violate physical principles, this study introduces a Rule-Based Constraint Layer. This layer incorporates domain knowledge to enforce a set of rigid and soft constraints that adjust the outputs of the DWC.

For example, according to the law of mass conservation, gas concentration cannot abruptly increase in the absence of external inputs. Therefore, when condition $∆C_{t}>\delta_{c}$ is detected and ${valve_{-}hange}_{t}$= 0, the window length is forcibly extended to a maximum value $w_{max}$, which enables the model to review an extended historical context to verify potential anomalies. Similarly, when the ambient temperature $T_{t}<T_{threshold}$ reaches a certain threshold, the gas diffusion rate decreases, and system response slows. Under such conditions, the window length should be increased to capture slowly evolving trends, which are as specified below:

(15) $$w_{t}^{final}=\min(w_{\max},w_{t}+\lambda \cdot {\left(T_{threshold}-T_{t}\right)}^{+}),$$

where $\left(x{)}^{+}=\max(0,x\right)$, and λ is a tuning coefficient. These rules are embedded into the model in the form of differentiable functions, ensuring that the constraint process can participate in gradient-based backpropagation.

### 3.3. Design of the GL-TransLSTM Model with Multimodal Feature Coordination

The proposed GL-TransLSTM prediction network is a hybrid deep learning architecture designed for modeling highly noisy and non-stationary industrial time series, with the goal of achieving high-precision, robust, and interpretable predictions of gas pipeline leakage processes. The overall structure of the network consists of five core modules, forming a progressive processing pipeline of “signal decomposition → feature extraction → fusion prediction,” which fully integrates the advantages of signal preprocessing techniques and deep neural networks, significantly enhancing the model’s generalization capability and predictive stability under complex working conditions. The pseudocode for this Algorithm 1 is shown below:
**Algorithm 1**: **GL-TransLSTM for Gas Pipeline Leakage Prediction**
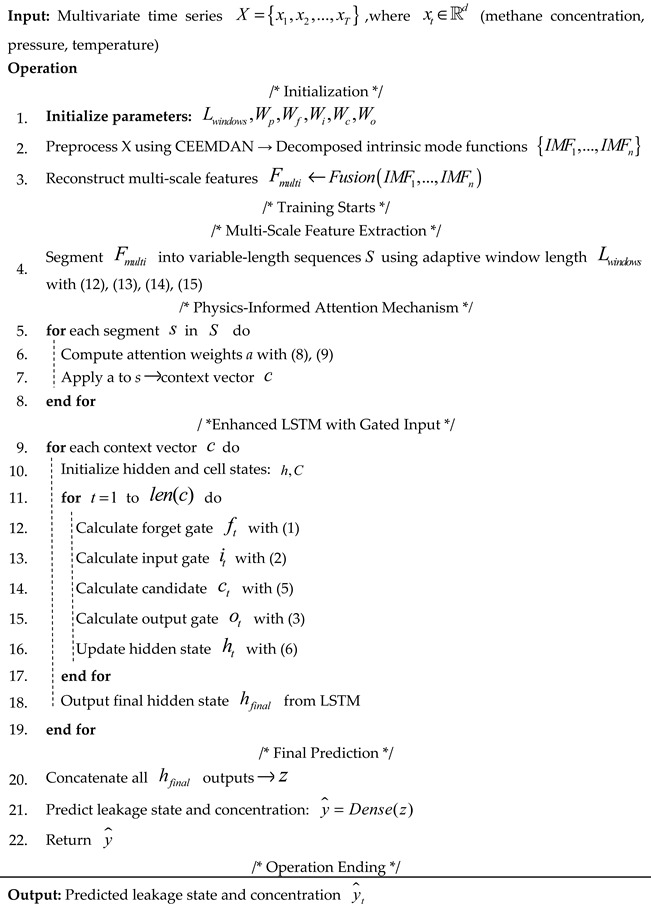


As shown in the pseudocode, firstly, the model receives raw time series signals from field-deployed sensors at the input layer, which covers multi-source heterogeneous data such as methane concentration, ambient temperature and humidity, pipeline pressure, flow rate and valve status. The temporal length of the input sequence can be flexibly determined based on specific application scenarios, thus typically following the system’s dynamic response requirements to ensure sufficient contextual information for trend inference.

Subsequently, to address the strong noise, nonlinearity and multi-scale fluctuations present in the raw signals, the model incorporates Complete Ensemble Empirical Mode Decomposition with Adaptive Noise (CEEMDAN) as a front-end signal decomposition layer. CEEMDAN adaptively decomposes the original non-stationary time series into several Intrinsic Mode Functions (IMFs) and a residual trend term, with each IMF representing a local oscillation mode at a different frequency scale. This decomposition process effectively separates high-frequency noise from low-frequency trends, achieving multi-modal decoupling of the signal. As a result, subsequent neural networks can perform differentiated modeling on the dynamic characteristics of each component, thereby improving overall prediction accuracy.

During the feature extraction stage, this study designs a GL-TransLSTM cooperative encoding mechanism to overcome the limitations of traditional single-model approaches. Specifically, each IMF component obtained from CEEMDAN decomposition is independently fed into an LSTM subnetwork to capture its local temporal dependencies. Thanks to its gated structure, the LSTM excels at modeling short-term dynamic variations in sequences, particularly in capturing nonlinear transitions between adjacent time steps, effectively compensating for the standard Transformer’s weakness in local detail modeling. To preserve temporal position information and support parallel computation, the model applies sinusoidal and cosine-based positional encoding to the hidden state sequences output by the LSTM, embedding temporal order information into the feature vectors. The encoded sequences are then input into a Transformer encoder, which leverages multi-head self-attention mechanisms to capture long-range dependencies across time steps, thus identifying potential periodic, trend-based, or anomalous evolution patterns.

In the feature fusion stage, the model employs a fully connected neural network layer to perform weighted fusion of the dual-path feature representations output by the LSTM and Transformer encoders. This fusion layer integrates both the local dynamic features extracted by the LSTM and the global semantic relationships captured by the Transformer, achieving complementary enhancement of local and global information. Additionally, residual connections and normalization operations are introduced during fusion to further improve training stability and gradient propagation efficiency.

Finally, the fused high-dimensional features are linearly mapped to generate predicted sequences for the corresponding IMF components at the output layer. The predictions of all components are summed according to CEEMDAN reconstruction rules to produce the final prediction output at the original signal scale. The decomposition–prediction–reconstruction strategy effectively reduces the complexity of the original series, enabling the model to adopt a divide-and-conquer approach and progressively approximate the true dynamics, significantly enhancing sensitivity to weak leakage signals and the ability to track long-term trends.

**Figure 3:**
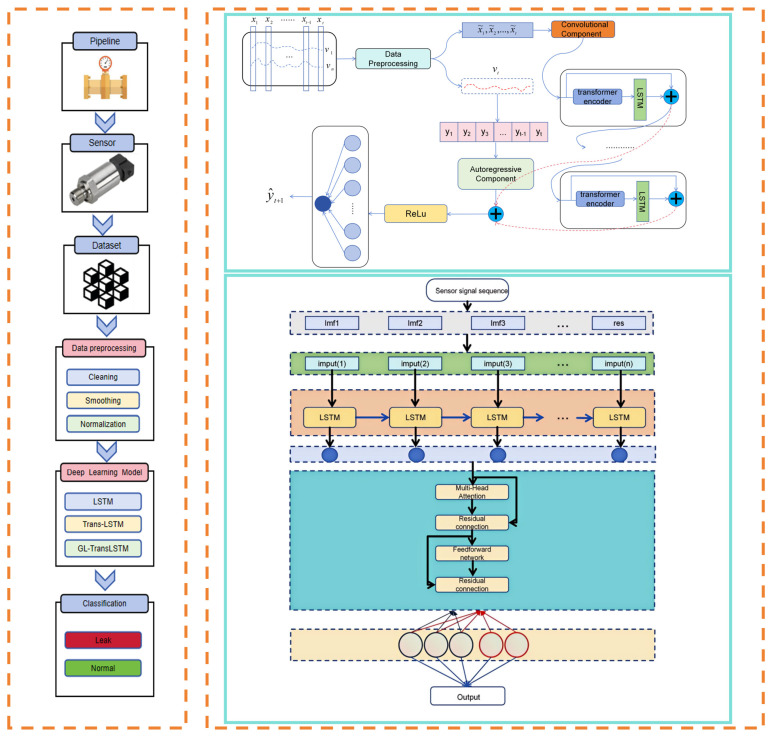
Architectural diagram of the GL-TransLSTM network model.

In summary, the GL-TransLSTM model employs a five-stage architecture, which comprises CEEMDAN-based signal preprocessing. LSTM-driven local feature extraction, positional encoding enhancement, transformer-enabled global modeling and multimodal fusion-reconstruction, which is used to form an intelligent prediction system that integrates temporal sensitivity, long-range dependency modeling, and noise robustness. This framework provides an efficient and reliable solution for industrial gas leakage monitoring.The network architecture diagram of GL TransLSTM is shown in Figure 3:

## 4. Experiments and Analysis

### 4.1. Dataset

**Figure 4:**
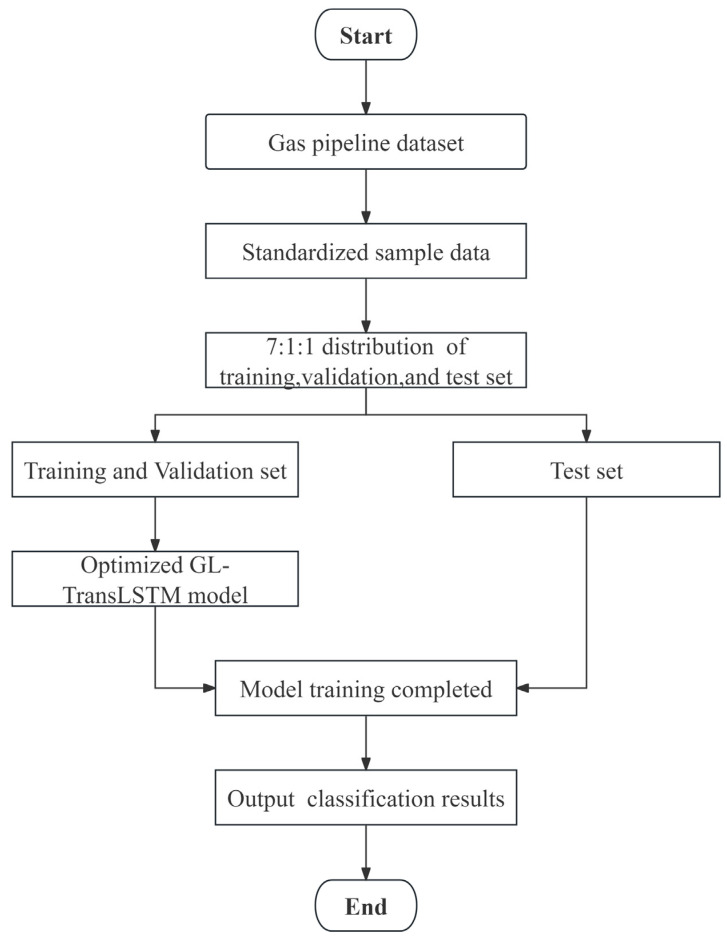
Flowchart of the data collection process.

In this study, data collection is conducted under normal and leakage conditions of the pipeline system to construct a comprehensive experimental dataset. During normal operation, the control valve remains closed, and the pipeline operates at a set temperature of 24.5 °C with a pressure of 0.3 MPa. The sampling interval is 5 min, and the gas flow rate is 60 m^3^·h^−1^. The data acquisition procedure is as follows: Firstly, we close the fluid control valve (i.e., leakage point) and start gas delivery. Then we record data continuously for 12 months while maintaining pipeline pressure at 0.3 MPa, which represents the dataset under normal conditions. After completing the normal state data collection, we adjust the control valve opening to 0.5 mm and continue recording data for 2 days. This dataset represents pressure, temperature, and concentration changes under leakage conditions. The entire switching process is continuous to ensure uninterrupted data collection. By repeating this process, two datasets with leakage apertures of 0.5 mm and 1.0 mm are completed. The data collection process is shown in Figure 4:

### 4.2. Parameters and Operating Environment

The dataset used in this study contains 120,000 samples. In deep learning (DL), key hyper-parameters include the number of training epochs (i.e., iterations over the training data), batch size (the number of samples processed before updating model parameters), and learning rate (the step size for weight adjustments). Experimental results demonstrate that fine-tuning these parameters leads to a significant improvement in classification performance.

The data is partitioned sequentially in chronological order, with 70%, 10%, and 10% allocated to the training, validation, and test sets, respectively. The remaining 10% of the data are discarded to prevent adjacent segments from appearing simultaneously in both training and test sets, thereby ensuring no information leakage across splits. To ensure a fair comparison, three consecutive deep learning models are constructed with identical architectures and hyper-parameter configurations. All models are optimized using the Adam algorithm and employ categorical cross-entropy as the loss function for the multi-label classification task. A dropout rate of 0.2 is applied in all models to mitigate overfitting. Training is terminated after 100 epochs, and the model parameters achieving the highest validation accuracy are retained for final evaluation on the test set. Detailed hyper-parameter settings are provided in the Table 2 and Table 3 below, and Table 4 provides our experimental environment:

### 4.3. Evaluation Metrics

To comprehensively evaluate the performance of the adopted models, LSTM, Transformer-LSTM, CNN, GRU, Transformer, BPNN, and GL-TransLSTM, we computed eight key metrics: accuracy (*Acc*), precision (*Pre*), F1-score (*F*1*score*), recall (*Rec*), mean squared error (*MSE*), mean absolute error (*MAE*), mean absolute percentage error (*MAPE*), and coefficient of determination (R^2^).

(16) $$Acc=\frac{TP+TN}{TP+TN+FP+FN},$$

(17) $$Pre=\frac{TP}{TP+FP},$$

(18) $$Recall=\frac{TP}{TP+FN},$$

(19) $$F1score=\frac{2.Pre.Rec}{Pre+Rec},$$

(20) $$MSE\left(y_{i},{\hat{y}}_{i}\right)=\frac{1}{n}{\sum_{i=1}^{n}\left(y_{i}-{\hat{y}}_{i}\right)}^{2},$$

(21) $$MAE(y_{i},{\hat{y}}_{i})=\frac{1}{n}\sum_{i=1}^{n}\left|y_{i}-{\hat{y}}_{i}\right|,$$

(22) $$MAPE\left(y_{i},{\hat{y}}_{i}\right)=\frac{100\%}{n}\sum_{i=1}^{n}\left|\frac{\left(y_{i}-{\hat{y}}_{i}\right)}{y_{i}}\right|,$$

where *TP*, *TN*, *FP*, and *FN* denote true positive, true negative, false positive, and false negative labels, respectively. $y_{i}$ denotes the actual value at the *i*-th time step, ${\hat{y}}_{i}$ denotes the predicted value at the *i*-th time step, $\bar{y}$ represents the overall mean value of the sequence data, *n* denotes the size of the test set, *Pre* denotes precision, *Rec* denotes recall.

### 4.4. Results and Discussion

This study proposes a pipeline leakage detection and analysis method based on the GL-TransLSTM model. In contrast to traditional approaches that often rely on manual inspection and visual examination, the proposed solution employs a time series deep learning framework to achieve more efficient and safer real-time monitoring. A detection system integrating physical techniques, sliding window processing, and time series deep learning algorithms is constructed to identify leakage conditions by analyzing subtle variations in concentration signal sequences. To process these signals, the system utilizes multiple deep learning models, LSTM, Transformer-LSTM, CNN, GRU, Transformer, BPNN, and GL-TransLSTM, to classify the leakage status into three categories: normal operation, minor leakage, and moderate leakage.

As mentioned earlier, seven deep learning models, LSTM, Transformer-LSTM, CNN, GRU, Transformer, BPNN, and GL-TransLSTM are applied to pipeline leakage classification. All models share identical structures, architectures, and hyper-parameter configurations to ensure a fair and optimal performance comparison. Model performance is evaluated using convergence curves, confusion matrices, and accuracy, supplemented by additional metrics including precision, F1-score, and recall.

**Figure 5:**
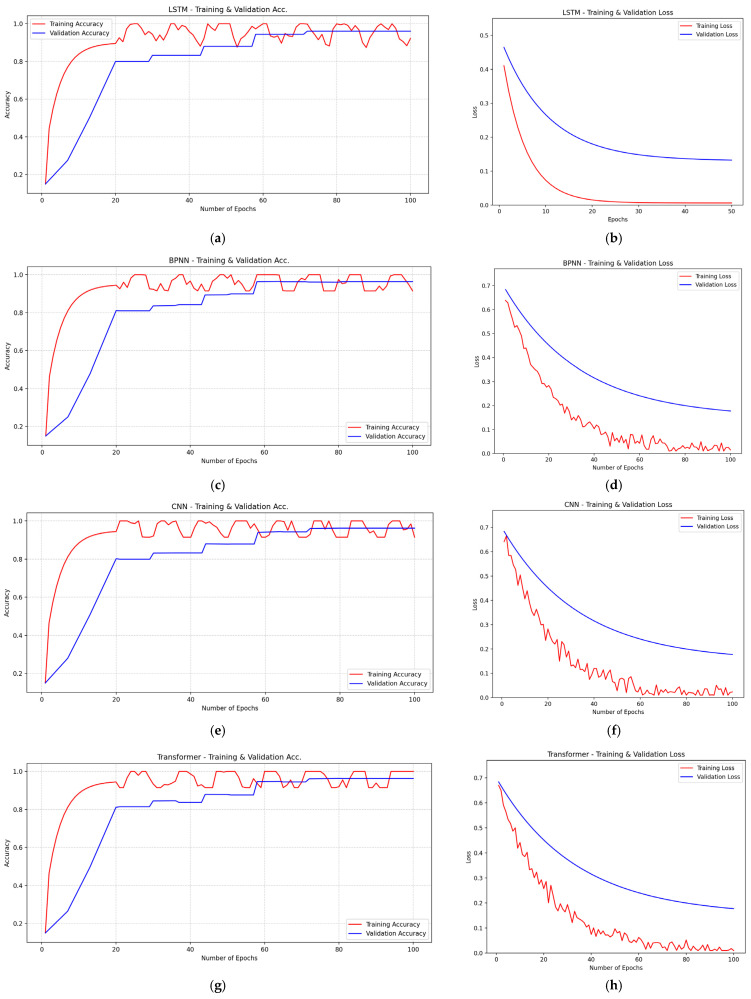

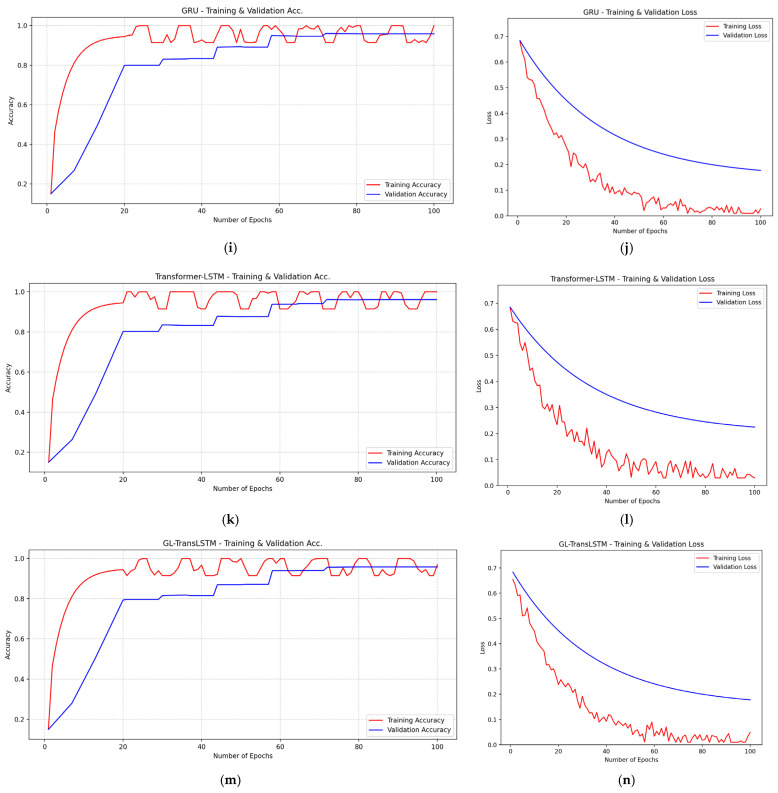
Accuracy and Loss Curves: (**a**) Accuracy of the LSTM model. (**b**) Loss of the LSTM model. (**c**) Accuracy of the BPNN model. (**d**) Loss of the BPNN model; (**e**) Accuracy of the CNN model. (**f**) Loss of the CNN model. (**g**) Accuracy of the Transformer model. (**h**) Loss of the Transformer model. (**i**) Accuracy of the GRU model. (**j**) Loss of the GRU model. (**k**) Accuracy of the Transformer-LSTM model. (**l**) Loss of the Transformer-LSTM model. (**m**) Accuracy of the GL-TransLSTM model. (**n**) Loss of the GL-TransLSTM model.

By varying the number of training epochs (as shown in Figure 5), the accuracy and loss convergence curves of the seven models are assessed. A detailed analysis of the training and validation accuracy curves reveals that although all models achieve high accuracy, the differences among them are minimal. A training duration of 50 epochs is deemed sufficient to evaluate the accuracy and loss characteristics of the models. When trained for 10 to 20 epochs, all seven models maintain accuracy between 80% and 90% across the training, testing, and validation sets. As the number of epochs increases from 20 to 40, accuracy remains within the 90% to 95% range. When approaching 80 epochs, model accuracy improves significantly, reaching 96% to 100%. Visualization of the accuracy trends indicates that all models reach peak accuracy with strong agreement between the training and validation data. All models are trained for 100 epochs, and both accuracy and loss values for the training and validation sets are recorded. Notably, at 100 training epochs, the loss values for these models fluctuate within a margin of less than 0.10. Thus, loss analysis confirms that all models achieve very low error rates after successful training.

To systematically evaluate the performance of various models in gas pipeline leakage detection, this study conducts a comprehensive comparative analysis of seven deep learning models, LSTM, BPNN, CNN, Transformer, GRU, Transformer-LSTM and the proposed GL-TransLSTM, across different batch sizes, which can be assessed by precision, recall, and F1-score (as shown in Table 5). Experimental results demonstrate that GL-TransLSTM achieves the best performance on all metrics such as precision, recall, and F1-score reaching 99.93%, 99.86%, and 99.89%, respectively.

Specifically, compared to the strongest baseline model Transformer-LSTM, GL-TransLSTM improves precision by 1.1%, recall by 0.98%, and F1-score by 1.01%. When compared to the classical LSTM model, it achieves more substantial improvements of 4.15%, 3.04%, and 2.11%, respectively. All other models, BPNN, CNN, Transformer, and Transformer-LSTM, are also outperformed by GL-TransLSTM across every metric. These results confirm that GL-TransLSTM maintains high precision and obtains superior recall, thus achieving an optimal balance between minimizing missed detections and suppressing false alarms.

The performance advantages are attributed to the deeply integrated physical prior knowledge and adaptive mechanisms within the model architecture. The Physics-Aware Gated Attention (PAGA) mechanism incorporates physical variables, such as ambient temperature and pressure gradients, as gating signals to dynamically adjust self-attention weights, significantly enhancing the model’s sensitivity to transient signal features caused by leakage events. The Physics-Guided Adaptive Sliding Window (PG-ASW) strategy dynamically adapts sampling window length and feature weights according to real-time operational conditions, effectively overcoming multi-scale representation challenges posed by non-stationary signals and improving the model’s generalization under complex working environments. Furthermore, by coupling LSTM’s capacity for capturing local dynamic features with the enhanced Transformer’s strength in modeling long-range dependencies, and incorporating residual connections and feature concatenation, the model achieves synergistic modeling of fine local variations and global temporal patterns, substantially improving both the representational robustness and classification accuracy for early leakage characteristics.

**Figure 6:**
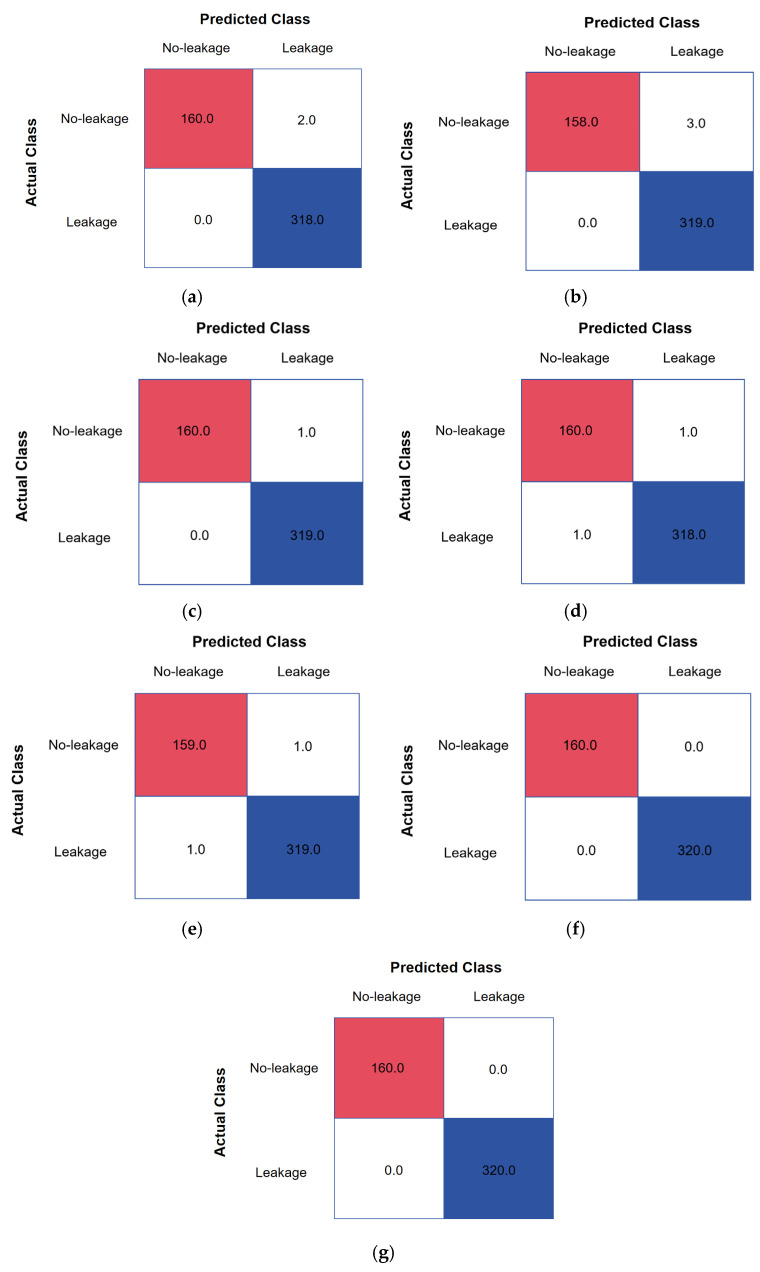
Confusion matrices for leakage detection: (**a**) LSTM; (**b**) BPNN; (**c**) CNN; (**d**) Transformer; (**e**) GRU; (**f**) Transformer-LSTM; (**g**) GL-TransLSTM.

**Figure 7:**
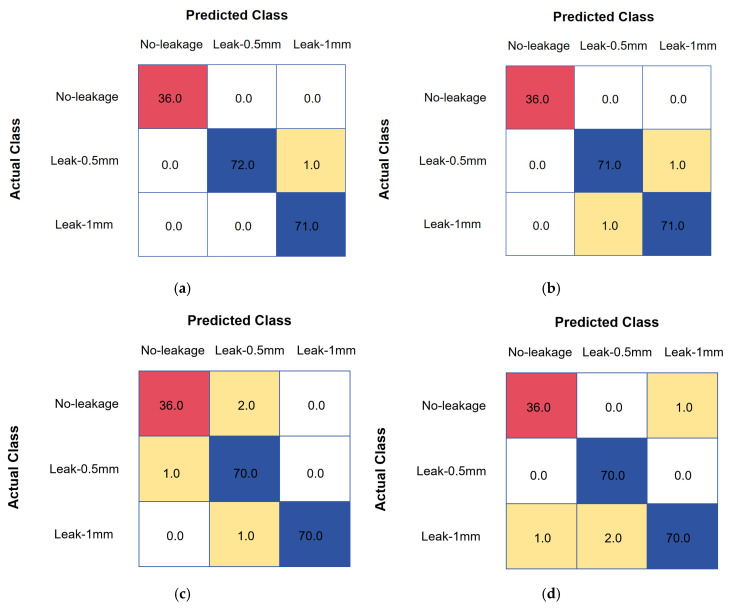

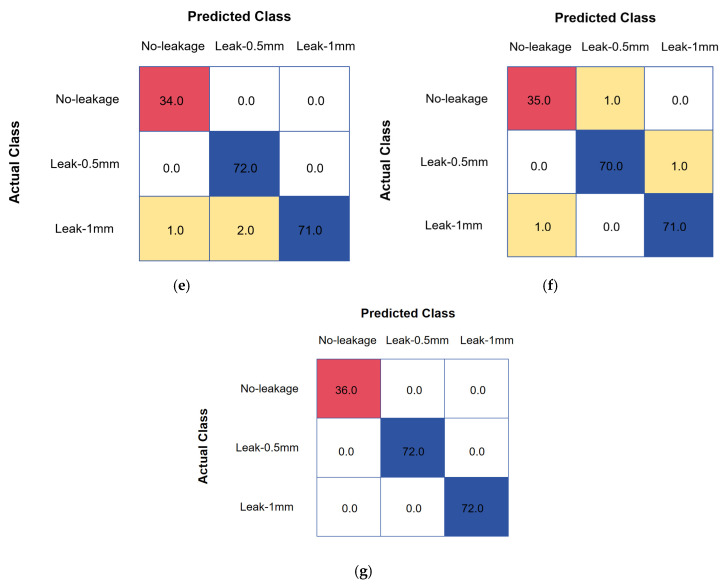
Confusion matrices for leakage size identification: (**a**) LSTM model for 0.5 mm and 1.0 mm leakage sizes; (**b**) BPNN model for 0.5 mm and 1.0 mm leakage sizes; (**c**) CNN model for 0.5 mm and 1.0 mm leakage sizes; (**d**) Transformer model for 0.5 mm and 1.0 mm leakage sizes; (**e**) GRU model for 0.5 mm and 1.0 mm leakage sizes; (**f**) Transformer-LSTM model for 0.5 mm and 1.0 mm leakage sizes; (**g**) GL-TransLSTM model for 0.5 mm and 1.0 mm leakage sizes.

Notably, the superiority of GL-TransLSTM is evident in binary classification tasks and the more complex task of leakage size identification. As shown in Figure 6 and Figure 7, this study compares the classification performance of different models across multiple leakage sizes (No-leakage, Leak-0.5 mm, Leak-1 mm); GL-TransLSTM consistently achieves higher recognition accuracy under all size conditions when compared to the baseline models.

In this study, we systematically evaluate the performance of multiple deep learning models, including LSTM, BPNN, CNN, Transformer, GRU, Transformer-LSTM, and GL-TransLSTM, in concentration prediction tasks. By comparing the fitting degree between predicted results and actual values on both training and test sets, we find that the GL-TransLSTM model demonstrates the best performance among all evaluated models.

Figure 5a–h display the prediction performance of LSTM, BPNN, CNN, and Transformer models on the training and test sets. These figures indicate that although these models can capture general concentration trends to some extent, they exhibit certain limitations in handling complex fluctuations and extreme values. For example, the LSTM model (Figure 5a,b) performs adequately on the training set but shows noticeable deviations on the test set, particularly in high-concentration peak regions. The BPNN model (Figure 5c,d), despite good fitting on the training set, demonstrates limited generalization on the test set, especially in regions with sharp concentration changes where predictions fail to fully replicate actual trends. The CNN model (Figure 5e,f) underperforms in processing time series data, with significant gaps between predictions and actual values, particularly in low-concentration regions. The Transformer model (Figure 5g,h), while fitting well in certain intervals, lags behind other models overall, especially in long-sequence predictions where accuracy decreases.

In contrast, the GL-TransLSTM model (Figure 5m,n) exhibits outstanding performance on both training and test sets. It demonstrates excellent fitting capability on the training set and accurately captures actual value trends on the test set, particularly in handling complex concentration variations and extreme values, where its predictions closely align with true values. This indicates strong stability and reliability in concentration prediction tasks. Specifically, on the training set, the prediction curve of GL-TransLSTM almost perfectly reproduces actual concentration changes, including accurate capture of high-concentration peaks. On the test set, even with unseen data, the model maintains high prediction accuracy, with minimal deviation between predicted and actual values, highlighting its superior generalization ability and robustness.

By comparing the mean squared error (MSE) of different models across the training, validation, and test sets, GL-TransLSTM demonstrates superior and consistently robust performance. As shown in Table 6, GL-TransLSTM achieves the lowest MSE values of 0.0022, 0.0027, and 0.0024 on the training, validation, and test sets, respectively. In contrast, the baseline Transformer-LSTM model yields MSEs of 0.0036, 0.0033, and 0.0035, indicating that the integration of PAGA, PG-ASW, and CEEMDAN in GL-TransLSTM effectively enhances both fitting capacity and generalization. All other conventional models, LSTM (0.029, 0.023, 0.026), BPNN (0.0038, 0.045, 0.041), CNN (0.0051, 0.046, 0.048), Transformer (0.0040, 0.051, 0.043), and GRU (0.0038, 0.032, 0.040), exhibit significantly higher errors, particularly on the validation and test sets, reflecting their limited ability to model complex, physics-informed temporal dynamics. Notably, GL-TransLSTM maintains a minimal performance gap between training and test phases, confirming its strong generalization and resistance to overfitting. These results collectively validate the efficacy of the proposed architecture in achieving high-precision leakage prediction under real-world industrial conditions.

In terms of mean absolute error (MAE), as presented in Table 7, GL-TransLSTM achieves a value of 0.0493 on the test set, significantly outperforming LSTM (0.0715), BPNN (0.0946), CNN (0.0538), Transformer (0.0829), GRU (0.0671), and Transformer-LSTM (0.0756). Specifically, GL-TransLSTM reduces the absolute error by over 3% compared to LSTM, CNN, and GRU, by approximately 4.5% compared to BPNN, by 3.36% compared to Transformer, and by 2.63% compared to Transformer-LSTM, demonstrating superior prediction stability and robustness. These findings further validate its leading performance in point estimation tasks.

Finally, regarding the mean absolute percentage error (MAPE), Table 8 shows that GL-TransLSTM attains an exceptionally high goodness-of-fit of 0.1201 on the test set, substantially surpassing LSTM (0.1912), BPNN (0.1457), CNN (0.1763), Transformer (0.1329), GRU (0.1084) and Transformer-LSTM (0.1601). Its MAPE value is approximately 4% higher than that of the second-best model, Transformer-LSTM, and over 5% higher than traditional LSTM and CNN. Collectively, these three metrics confirm that GL-TransLSTM achieves optimal performance in both error control and model interpretability, thus fully underscoring the effectiveness and superiority of its architectural design.

Through comparative experiments and visual analysis, this section thoroughly validates the outstanding performance of the GL-TransLSTM model in gas pipeline leakage concentration prediction. By integrating the local temporal modeling capability of Long Short-Term Memory (LSTM) networks and the global dependency capture mechanism of an enhanced Transformer architecture, along with the incorporation of a physics-aware gated attention mechanism and an adaptive sliding window strategy, the model achieves high-precision, highly interpretable, and well-generalized predictions of complex dynamic processes. Its successful application provides efficient technical support for safety monitoring of gas pipelines and also offers a valuable modeling paradigm for other physically constrained time series prediction tasks such as equipment fault diagnosis and environmental monitoring.

### 4.5. Ablation Study

To systematically evaluate the contribution of each key component in the proposed GL-TransLSTM model, a series of ablation experiments are conducted. These experiments progressively remove or replace core modules, including the Physics-Aware Gated Attention (PAGA), the Physics-Guided Adaptive Sliding Window (PG-ASW), and the CEEMDAN based multi-scale feature decomposition, to quantitatively assess their individual and collective impact on leakage prediction accuracy, robustness, and generalization capability. By comparing the performance of various model variants under identical experimental conditions, this study not only validates the effectiveness of each proposed innovation but also elucidates the synergistic benefits of multimodal fusion and physics-informed learning in modeling non-stationary, high-noise industrial time series data. The results of the ablation experiment are shown in Table 9.

The ablation study results demonstrate that each proposed component contributes positively to the overall performance of GL-TransLSTM with the full model (base+PAGA+CEEMDAN+PG-ASW), which achieves the lowest MAE and MSE across all datasets. Specifically, PAGA yields the most significant individual improvement, reducing test MAE from 0.0756 (base) to 0.0648, which highlights the effectiveness of physics-aware attention in capturing relevant spatiotemporal dependencies. PG-ASW also provides consistent gains, while CEEMDAN alone slightly degrades performance, likely due to increased noise or redundancy in decomposed components without complementary mechanisms. However, when combined, all three modules exhibit strong synergy, with the full model further lowering test MAE to 0.0493, thus confirming that physics-guided attention, adaptive windowing and multi-scale decomposition jointly enhance prediction accuracy and generalization.

### 4.6. Comparative Experiment

To verify that the high performance of GL-TransLSTM is robust and not an artifact of carefully cherry-picked hyper-parameters, we conduct a sensitivity analysis by varying key hyper-parameters around their default settings while keeping all others fixed. As shown in Table 10, the model exhibits remarkable stability: most adjustments lead to only marginal changes in accuracy (ranging from 99.63% to 99.93%) and mean squared error (MSE between 0.0022 and 0.0035). Notably, even suboptimal choices—such as reducing the number of encoder layers to 1 or increasing the learning rate to 0.005—still yield accuracy above 98.6% and low reconstruction error. The fact that performance remains consistently high across diverse configurations indicates that the reported results reflect genuine model effectiveness rather than overfitting to a narrow set of hyper-parameters. This robustness underscores the reliability of GL-TransLSTM for real-world gas leakage detection under practical deployment constraints.

## 5. Conclusions

This study addresses critical challenges in gas pipeline leakage prediction, including high noise, non-stationarity, and multi-variable coupling, proposing a physics-informed deep learning model named GL-TransLSTM. The proposed model systematically integrates the local temporal modeling capability of Long Short-Term Memory (LSTM) networks with the global dependency capture mechanism of an enhanced Transformer architecture. By incorporating a physics-aware gated attention mechanism and an adaptive sliding window strategy, it demonstrates outstanding performance in both leakage detection and size identification tasks.

**Figure 8:**
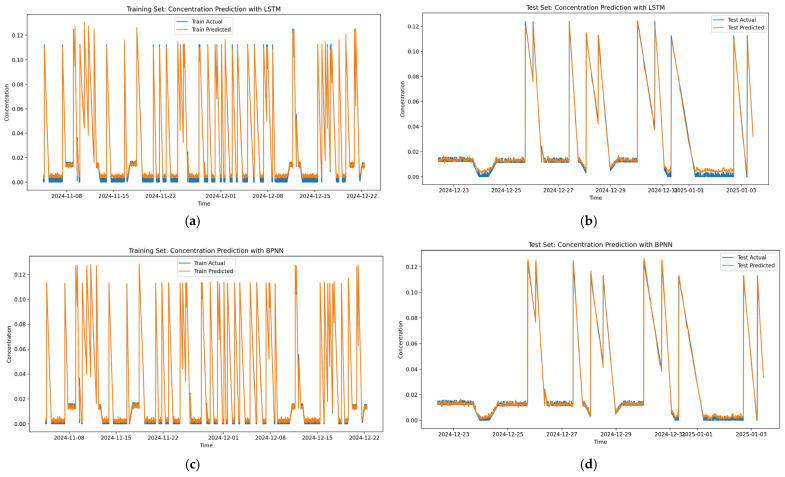

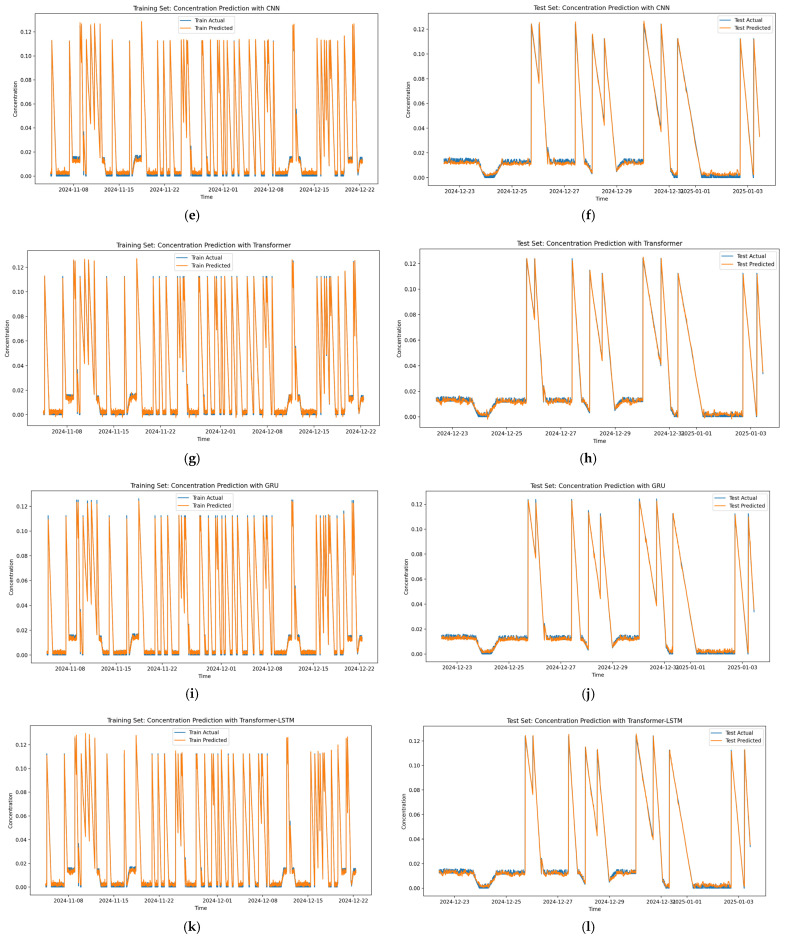

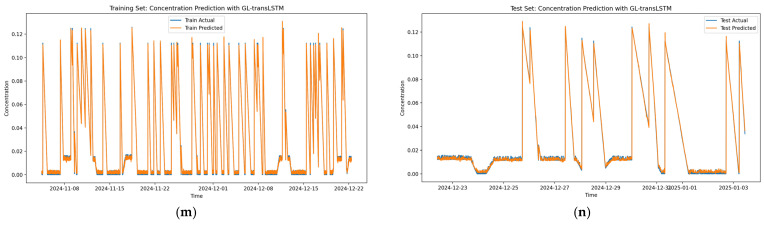
Prediction sequences: (**a**) training sequence of the LSTM model, (**b**) test sequence of the LSTM model, (**c**) training sequence of the BPNN model, (**d**) test sequence of the BPNN model, (**e**) training sequence of the CNN model, (**f**) test sequence of the CNN model, (**g**) training sequence of the Transformer model, (**h**) test sequence of the Transformer model, (**i**) training sequence of the GRU model, (**j**) test sequence of the GRU model, (**k**) training sequence of the Transformer-LSTM model, (**l**) test sequence of the Transformer-LSTM model, (**m**) training sequence of the GL-TransLSTM model, (**n**) test sequence of the GL-TransLSTM model.

The core strength of GL-TransLSTM lies in its deep integration of physical mechanisms with data-driven modeling. First, the introduction of the Physics-Aware Gated Attention (PAGA) mechanism enables the model to utilize physical variables such as ambient temperature and pressure gradients as dynamic conditioning signals, guiding self-attention weights to focus on time segments highly correlated with leakage events. This design shifts the model from purely statistical correlation towards causal reasoning, allowing it to automatically emphasize contextual information during key events such as valve operations or sudden pressure changes, thereby improving sensitivity to early weak leakage signals. Second, the Physics-Guided Adaptive Sliding Window (PG-ASW) strategy effectively overcomes the limitations of traditional fixed-length windows in handling multi-scale dynamic processes. By dynamically adjusting the input sequence length and feature weights according to current operating conditions and incorporating a multi-scale feature pyramid, the model achieves cross-scale information fusion, enabling flexible adaptation to complex variations ranging from second-level transients to hour-level slow drifts. This approach significantly improves the physical consistency and discriminative power of the feature representations. Furthermore, GL-TransLSTM demonstrates exceptional performance in leakage size identification. Visualization analyses in Figure 8 indicate that the model clearly distinguishes between different leakage severity levels with extracted feature spaces, which shows stronger intra-class compactness and inter-class separation. It allows the model to detect whether a leakage occurs, which also can accurately estimate its severity, providing more actionable information for operational decision-making. It is achieved through a multi-task joint loss function that simultaneously optimizes concentration prediction and state classification objectives during training, thus enhancing both sensitivity to anomalous patterns and generalization capability.

In summary, GL-TransLSTM successfully establishes an intelligent leakage prediction system through architectural innovation and the embedding of physical priors, which acquires high accuracy, strong interpretability, and excellent generalization. Its successful application offers an efficient technical solution for safety monitoring of gas pipelines, thus providing a valuable modeling paradigm for other physically constrained time series prediction tasks, such as equipment fault diagnosis and environmental monitoring. In particular, its Physics-Aware Gated Attention (PAGA) and Physics-Guided Adaptive Sliding Window (PG-ASW) modules embody a generalizable paradigm for integrating domain-specific physical constraints into deep time series models. The core idea is to transform interpretable physical variables or derived quantities (e.g., gradients, rates of change, conservation laws) into dynamic control signals that modulate attention weights or temporal windowing strategies.

This approach can be systematically extended to other domains: In power consumption forecasting, physical priors such as load balance equations, thermal inertia of buildings, or appliance duty cycles can serve as gating signals to highlight periods of high uncertainty or regime shifts. In weather prediction, thermodynamic constraints (e.g., Clausius–Clapeyron relation for humidity, geostrophic balance for wind fields) can inform attention regularization or adaptive temporal resolution.

We thus advocate for a modular design principle: (1) identify governing physical laws or empirical constraints in the target domain; (2) encode them as differentiable or learnable gating/regularization terms; (3) embed them into the attention or windowing mechanism of a hybrid sequence model. This “physics-as-control-signal” framework offers a pathway toward robust, interpretable, and data-efficient forecasting across diverse scientific and engineering applications. Future work will explore the potential of this model in multi-source heterogeneous sensor fusion, online incremental learning, and edge computing deployment, thus promoting the development of smarter and more autonomous intelligent maintenance systems.

## Figures and Tables

**Table 1:** Pipeline leak detection methods.

Study No.	Technology Used	Dataset	Evaluation Metric	Advantages	Limitations
[24]	CNN-SVM	Gas Pipeline Concentration Time Series	Acc MSE RMSE	By combining convolutional feature extraction with SVM classification, this hybrid model achieves high leak recognition accuracy.	It does not explicitly model temporal dynamics and overlooks the influence of varying flow regimes on leak signatures.
[25]	CNN-TL	Mixed dataset AW-AS	Acc MSE RMSE F1-Score	CNN-TL demonstrates strong adaptability across multiple operating conditions by integrating transfer learning or temporal learning strategies into the CNN framework.	It still inherits the inherent limitation of CNNs in capturing long-range temporal dependencies in time series pipeline data.
[26]	1-D CNN	Gas Pipeline Concentration Time Series	Acc MSE RMSE AUC-ROC	The one-dimensional CNN enables direct end-to-end estimation of leak size from raw sensor signals without manual feature engineering, achieving high identification accuracy.	The approach does not consider how different flow modes (e.g., laminar vs. turbulent) affect pressure dynamics, which may reduce generalization in real pipelines.
[27]	GAN-LSTM	Collect data in an oil Group	Acc MSE RMSE R^2^	GAN-LSTM effectively captures long-term temporal dependencies in sequential pipeline data and has demonstrated stable leak detection accuracy above 90% in field applications.	It primarily focuses on temporal patterns and often underutilizes spatial or multi-sensor correlations, limiting its ability to fuse heterogeneous features.
[29]	LSS model	NASA’s prognostics data repository	Acc MSE RMSE Rec	The LSS model enhances predictive performance by fusing LSTM’s sequential modeling with statistical process monitoring, significantly outperforming traditional RNNs and regression methods in degradation forecasting.	It is specifically designed for aero-engine bearing health monitoring and has not been validated for pipeline leakage scenarios, raising concerns about domain transferability.
[31]	BiLSTM	Data acquired from industrial site	Acc MSE RMSE Rec F1-Score	BiLSTM improves leak discrimination by modeling contextual information from both past and future time steps, effectively distinguishing real leaks from common pressure transients.	It may overlook high-frequency or localized signal characteristics, potentially leading to the loss of critical diagnostic information.

**Table 2:** Hyper-parameter settings of the GL-TransLSTM model.

Parameter Name	Parameter Value
LSTM Layers	2
Encoder Layers	2
Optimizer	Adam
Number of Heads	4
Full Connection Layer Hidden Unit	64
Learning Rate	0.001
Epoch	100

**Table 3:** Hyper-parameter configuration of the LSTM model.

Parameter Name	Parameter Value
Activation Function	tanh
Time Step	20
Optimizer	Adam
Loss Function	Cross-Entropy Loss
Number of Memory Units	50
Learning Rate	0.001
Epoch	100
Discard Layer Threshold	0.2

**Table 4:** Experimental environment for GL TransLSTM.

Category	Version
Operating System	Ubuntu 20.04 LTS/Windows 10
Programming Language	Python 3.8
Deep Learning Framework	PyTorch 1.9.1
GPU	NVIDIA GeForce RTX 4060 (8 GB VRAM)
CUDA Version	10.2
CPU	Intel Core i7-9700 (8 cores, 3.0 GHz)
System Memory	16 GB DDR4 RAM

**Table 5:** Performance summary of the proposed model under different batch sizes (BS) (BS 1 = 64, BS 2 = 128, BS 3 = 256).

Models	Labels	Precision			Recall			F1 Score		
		BS 1	BS 2	BS 3	BS 1	BS 2	BS 3	BS 1	BS 2	BS 3
LSTM	Leak	95.68	95.72	95.78	95.60	95.67	96.74	95.63	96.73	97.78
	Non-leak	95.73	95.65	95.68	95.57	95.74	96.82	96.61	95.64	95.83
BPNN	Leak	96.72	96.81	97.67	95.73	96.78	96.83	96.72	96.75	97.85
	Non-leak	96.81	96.76	96.83	96.79	96.83	96.85	96.83	96.85	96.88
CNN	Leak	95.83	95.86	95.89	95.79	95.85	95.88	95.82	95.86	95.92
	Non-leak	95.81	95.83	95.88	95.85	95.86	95.92	95.81	95.84	95.94
Transformer	Leak	97.82	97.85	97.87	97.79	97.83	97.85	97.84	97.85	97.89
	Non-leak	97.88	97.89	97.91	97.86	97.88	97.93	97.83	97.85	97.95
GRU	Leak	97.83	97.92	97.88	97.80	97.83	97.82	97.89	97.93	97.96
	Non-leak	97.76	97.83	97.89	97.82	97.84	97.89	97.88	97.95	97.98
Transformer-LSTM	Leak	98.67	98.72	98.83	98.62	98.66	98.76	98.65	98.69	98.75
	Non-leak	98.71	98.73	98.75	98.82	98.85	98.88	98.77	98.79	98.88
GL-TransLSTM	Leak	99.87	99.89	99.93	99.60	99.78	99.83	99.73	99.75	99.87
	Non-leak	99.82	99.73	99.85	99.76	99.83	99.86	99.78	99.85	99.89

**Table 6:** Comparison of MSE across different models.

Model	Training Set	Validation Set	Test Set
LSTM	0.029	0.023	0.026
BPNN	0.0038	0.045	0.041
CNN	0.0051	0.046	0.048
Transformer	0.0040	0.051	0.043
GRU	0.0038	0.032	0.040
Transformer-LSTM	0.0036	0.0033	0.0035
GL-TransLSTM	0.0022	0.0027	0.0024

**Table 7:** Comparison of MAE across different models.

Model	Training Set	Validation Set	Test Set
LSTM	0.0834	0.0927	0.0715
BPNN	0.0621	0.0583	0.0946
CNN	0.0912	0.0746	0.0538
Transformer	0.0753	0.0991	0.0829
GRU	0.0547	0.0632	0.0671
Transformer-LSTM	0.0886	0.0728	0.0756
GL-TransLSTM	0.0501	0.0508	0.0493

**Table 8:** Comparison of MAPE across different models.

Model	Training Set	Validation Set	Test Set
LSTM	0.1834	0.1275	0.1912
BPNN	0.1621	0.1836	0.1457
CNN	0.1158	0.1043	0.1763
Transformer	0.1947	0.1528	0.1329
GRU	0.1372	0.1991	0.1084
Transformer-LSTM	0.1203	0.1674	0.1601
GL-TransLSTM	0.1226	0.1107	0.1201

**Table 9:** Results of ablation study.

Model	Training Set		Validation Set		Test Set	
	MAE	MSE	MAE	MSE	MAE	MSE
Transformer-LSTM (base)	0.0886	0.0036	0.0728	0.0033	0.0756	0.0035
base+PAGA	0.0648	0.0025	0.0648	0.0029	0.0648	0.0026
base+PG-ASW	0.0726	0.0032	0.0726	0.0030	0.0726	0.0033
base+CEEMDAN	0.0843	0.0035	0.0843	0.0032	0.0843	0.0034
base+PAGA+PG-ASW	0.0586	0.0024	0.0586	0.0029	0.0586	0.0025
base+PAGA+CEEMDAN	0.0683	0.0025	0.0683	0.0028	0.0683	0.0026
base+PG-ASW+CEEMDAN	0.0715	0.0029	0.0715	0.0029	0.0715	0.0032
base+PAGA+CEEMDAN+PG-ASW	0.0501	0.0022	0.0508	0.0027	0.0493	0.0024

**Table 10:** Sensitivity analysis of GL-TransLSTM to key hyper-parameters.

Hyper-Parameter	Default Value	Adjusted Value	Acc (Default)	Acc (Adjusted)	MSE (Default)	MSE (Adjusted)
LSTM Layers	2	3	99.93	99.93	0.0024	0.0023
Encoder Layers	2	1	99.93	99.81	0.0024	0.0032
Number of Heads	4	8	99.93	99.92	0.0024	0.0029
FC Hidden Unit	64	128	99.93	99.91	0.0024	0.0022
Learning Rate	0.001	0.0005	99.93	98.86	0.0024	0.0035
Learning Rate	0.001	0.005	99.93	98.63	0.0024	0.0034
Epoch	100	150	99.93	99.92	0.0024	0.0025

## Data Availability

The data presented in this study are available on request from the corresponding author due to proprietary and confidentiality constraints, as the datasets are generated from internal corporate instrumentation and the associated code is executed on a secured internal computational platform.
